# TIGIT Blockade: A Multipronged Approach to Target the HIV Reservoir

**DOI:** 10.3389/fcimb.2020.00175

**Published:** 2020-05-05

**Authors:** Kayla A. Holder, Michael D. Grant

**Affiliations:** Immunology and Infectious Diseases Program, Division of BioMedical Sciences, Faculty of Medicine, Memorial University of Newfoundland, St. John's, NL, Canada

**Keywords:** HIV-1, TIGIT, PVR, checkpoint inhibitor, T cell, NK cell

## Abstract

During chronic human immunodeficiency virus type 1 (HIV-1) infection, upregulation of inhibitory molecules contributes to effector cell dysfunction and exhaustion. This, in combination with the ability of HIV-1 to reside dormant in cellular reservoirs and escape immune recognition, makes the pathway to HIV-1 cure particularly challenging. An idealized strategy to achieve HIV-1 cure proposes combined viral and immune activation by “shock”ing HIV-1 out of latency and into an immunologically visible state to be recognized and “kill”ed by immune effector cells. Here we outline the potential for blockade of the inhibitory immune checkpoint T cell immunoreceptor with immunoglobulin and ITIM domains (TIGIT) to overcome natural killer (NK) cell and T cell inhibition associated with HIV-1 infection and invigorate antiviral effector cell responses against HIV-1 reactivated from the latent cellular reservoir.

## Introduction

Combination antiretroviral therapy (cART) reduces human immunodeficiency virus type 1 (HIV-1) replication to levels where the amount of viral ribonucleic acid (RNA) in the bloodstream falls below current limits of detection. In most cases, maintenance of undetectable viral loads requires strict adherence to therapy (Chun et al., 1995, 1997; Finzi et al., 1999). Despite their efficacy, complete eradication of HIV-1 is unattainable with current cART regimes. During early infection, HIV-1 establishes proviral reservoirs, concealing itself within various cell types in different anatomical niches (Wong and Yukl, 2016; Baxter et al., 2018). In this largely dormant state, the HIV-1 reservoir is invisible to the immune system and insensitive to cART (Finzi et al., 1997). As a consequence of this widespread thorough concealment, if cART is interrupted, HIV-1 reactivates and produces replication-competent viruses capable of nascent infection (Wong et al., 1997; Finzi et al., 1999). Organs and tissues such as the gut and lymph nodes are key sites enriched for cells harboring HIV-1 provirus (Wong and Yukl, 2016). Although various types of cells including macrophages, monocytes and astrocytes can serve as HIV-1 reservoirs, the predominant cell type containing HIV-1 provirus are CD4^+^ T cells and, thus, they are the predominant source of viral replication with withdrawal of cART (Finzi et al., 1999; Wong et al., 2019). Seeding itself in long-lived memory CD4^+^ T cells during acute and ongoing infection allows HIV-1 to persist indefinitely, despite consistent and effective cART suppression.

In the absence of cART, activation of the resting CD4^+^ T cells harboring HIV-1 provirus drives HIV-1 out from latency, replenishes the reservoir and promotes disease progression. Cure of the “Berlin patient” in 2008 and the “London patient” in 2019 with HIV-1-resistant bone marrow transplants provides proof of concept that HIV-1 can be eradicated in those already living with the virus (Hutter et al., 2009; Gupta et al., 2019). Although application of this approach is not feasible for the vast majority of people living with HIV-1 (PLWH), other elimination strategies are under investigation. These can include “block and lock” or gene editing, both of which aim to fix latent proviral HIV in a permanent inactive state with either drug therapy or *in situ* HIV genome editing. Conversely, a “kick/shock and kill” approach focuses on purging the latent HIV-1 reservoir by forced HIV activation from reservoir cells, thereby exposing it to the immune system and/or cART (Deeks, 2012; Shan et al., 2012; Qu et al., 2013; Ahlenstiel et al., 2015; Mousseau et al., 2015; Zhu et al., 2015; Karpinski et al., 2016; Margolis et al., 2016). To completely cure HIV-1 infection by this latter approach, two currently unattainable objectives must be met. Firstly, viral reactivation needs to occur in all latently infected cells bearing replication competent viral genomes. Secondly, those cells in which HIV-1 reactivates must be eliminated efficiently enough to prevent spread to uninfected cells. The second goal requires enhanced antiviral immune function, likely combined with novel pharmacologic strategies. Direct reservoir cytolysis by T cell and specific antibody-dependent NK cell mechanisms is a key element of this goal. Incomplete purging of the latent HIV-1 reservoir, although not an absolute cure, may be sufficient to reduce or even remove dependence upon cART for suppression of HIV replication and yield a functional cure for HIV-1 infection. In light of the role that the immune system will play, similarities between cancer and chronic viral infection imply that administration of checkpoint inhibitors can benefit immune-based HIV-1 cure and treatment strategies.

Like cancer, chronic viral infection often progresses to a stage where effector cell functions fundamental for its control are severely impaired (Wherry and Kurachi, 2015; Bi and Tian, 2017). Following activation, T cells upregulate inhibitory receptors such as CTLA-4 and PD-1 to limit T cell responses and prevent immune pathology arising from unregulated responses (Wherry and Kurachi, 2015). In settings of chronic infection with persistent microbial replication, T cell function is dysregulated by sustained high expression of these inhibitory checkpoint receptors (Attanasio and Wherry, 2016; Wykes and Lewin, 2018). Checkpoint inhibitors targeting different inhibitory receptors on immune cells or their corresponding ligands are transforming cancer therapy and many are relevant to immunotherapy for HIV-1 infection. We focused this review on the T cell immunoreceptor with immunoglobulin and ITIM domains (TIGIT) immune checkpoint receptor as expression of TIGIT, its competitors, and its ligands are broadly dysregulated on multiple cell types in HIV-1 infection. Furthermore, recent studies indicate that TIGIT negatively regulates both T cell and NK cell antiviral effector functions. We will discuss findings that suggest that this regulatory axis is an especially exploitable immune checkpoint in HIV-1 reservoir elimination strategies engaging antiviral effector cells.

### Differential TIGIT Expression on Immune Cells

**Figure 1:**
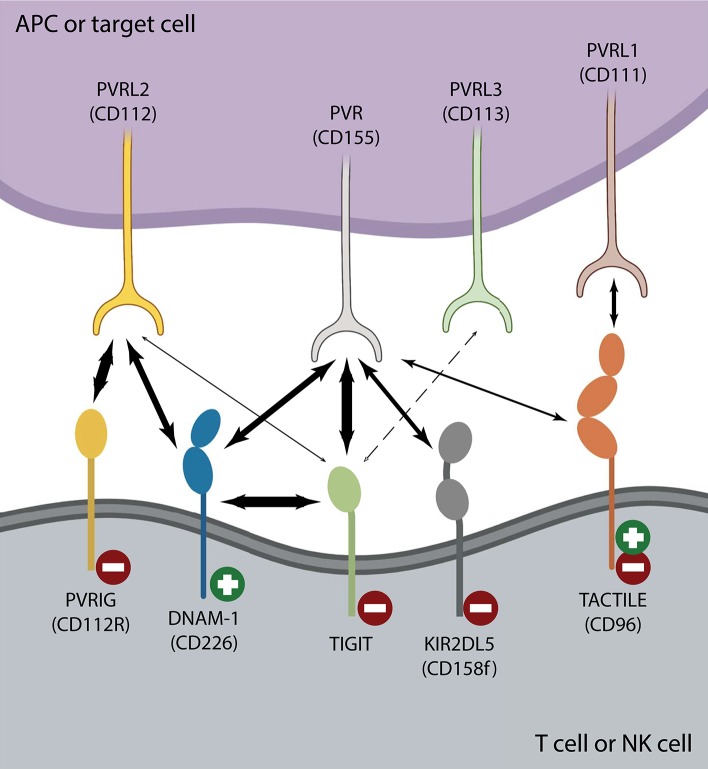
The TIGIT/DNAM-1 immune checkpoint axis. Interactions between inhibiting (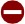) and activating (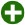) T cell or NK cell receptors belonging to the nectin or nectin-like family of receptors and their corresponding family of ligands are depicted. Strong interactions such as those between TIGIT and PVR or DNAM-1 in *cis* or PVRIG and PVRL2 are illustrated with heavy arrows. There is no clear consensus regarding whether TIGIT binds PVRL3 (dotted arrow) and it is unclear whether TIGIT/PVRL2 interactions are physiologically relevant *in vivo* (Stanietsky et al., 2009; Yu et al., 2009; Whelan et al., 2019). DNAM-1 interacts with both PVR and PVRL2 to counter inhibition, yet does so with lower affinity than either TIGIT or PVRIG. TACTILE preferentially interacts with PVRL1 over PVR (Holmes et al., 2019). The affinity of KIR2DL5 for PVR binding is currently unknown, as is whether any other nectin or nectin-like ligand or receptor can serve as its binding partner.

Most NK cells and multiple T cell subsets, including memory T cells, regulatory T cells and follicular helper T cells (T_FH_), express TIGIT (Boles et al., 2009; Stanietsky et al., 2009; Yu et al., 2009; Levin et al., 2011; Wang et al., 2015; Wu et al., 2016). After interaction with either of its ligands, poliovirus receptor (PVR or CD155 or Necl-5), or PVRL2 (CD112 or nectin-2), TIGIT inhibits activation of T cell or NK cell effector functions (Stanietsky et al., 2009; Yu et al., 2009; Stengel et al., 2012). TIGIT belongs to a larger family of nectin and nectin-like receptors that all recognize the same group of ligands (Chan et al., 2012; Pauken and Wherry, 2014). Like TIGIT, TACTILE (CD96), and PVR-related Ig domain (PVRIG or CD112R) bind PVR, and PVRL2, respectively, whereas DNAM-1 (CD226) is a costimulatory counter receptor that competes with both TIGIT and TACTILE for PVR engagement and with PVRIG for PVRL2 binding (Figure 1) (Anderson et al., 2016; Zhu et al., 2016; Dougall et al., 2017; Xu et al., 2017; Sanchez-Correa et al., 2019). The inhibitory receptor PVRIG is expressed on activated T cells and NK cells (Figure 1), however, there is a lack of conclusive evidence in human NK cell studies as to whether TACTILE negatively or positively regulates activation (Fuchs et al., 2004; Georgiev et al., 2018; Whelan et al., 2019). Although PVR is a common ligand for TIGIT, TACTILE, and DNAM-1, the binding affinities vastly differ, with TIGIT having a greater affinity for PVR than either DNAM-1 or TACTILE (Figure 1) (Yu et al., 2009). This domination TIGIT has over DNAM-1 for ligand binding favors effector cell inhibition over effector cell costimulation, thereby dampening immune responses. Another means by which TIGIT controls T cell or NK cell activation is by interfering with DNAM-1 homodimerization by forming a heterodimer with DNAM-1 in *cis* (Figure 1) (Johnston et al., 2014). The intracellular TIGIT/DNAM-1 complex prevents effective intercellular DNAM-1/ligand interactions and reduces effector cell costimulation. This family of paired receptors and ligands constitute a regulatory signaling pathway resembling that of CD28 and CTLA-4 with antagonistic effects conveyed through differential receptor binding of the same ligand (Martinet and Smyth, 2015).

One hallmark of chronic HIV-1 infection is disruption of normal lymphocyte functions, leading to signs and symptoms of immune exhaustion. This exhaustion profile is illustrated by increased expression of multiple inhibitory immune checkpoint molecules including PD-1, CTLA-4, TIM-3, and LAG-3 on CD8^+^ T cells and in some instances, on NK cells (Wherry et al., 2007; Anderson et al., 2016). In contrast to these well-characterized exhaustion markers, TIGIT is found to varying extents on NK cells and naïve CD8^+^ T cells and is further upregulated after activation (Yu et al., 2009). There is convincing evidence of a central role for TIGIT in control of CD8^+^ T cell maturation and exhaustion (Johnston et al., 2014). However, considering its parallel regulation of NK cell functions, targeting TIGIT with checkpoint inhibitors may have even greater implications for bolstering antiviral immunity than targeting PD-1 or CTLA-4. Of all lymphocyte subsets, NK cells have the highest fraction of cells constitutively expressing TIGIT receptors (Wang et al., 2015). Between 20 and 90% of resting NK cells express TIGIT and levels are increased by acute and chronic viral infections or cancers (Bi et al., 2014; Johnston et al., 2014; Wang et al., 2015; Zhang et al., 2018).

Targeting TIGIT is an especially attractive approach to incorporate into HIV-1 cure strategies as it impacts multiple functions of multiple types of effector cells. Its widespread expression on NK cells and CD8^+^ T cells enhances the likelihood of TIGIT blockade having a meaningful impact in the setting of chronic infection. In this setting, CD8^+^ T cells acquire expression of inhibitory receptors, including TIGIT, all contributing to maintenance of an immune exhausted state. Utilizing therapeutic monoclonal antibodies (mAb) to release the brakes on exhausted CD8^+^ T cells and on NK cells expressing high amounts of TIGIT can counter inhibition to favor restoration of productive antiviral effector functions.

### TIGIT Regulates Effector Cells in HIV-1 Infection

**Figure 2:**
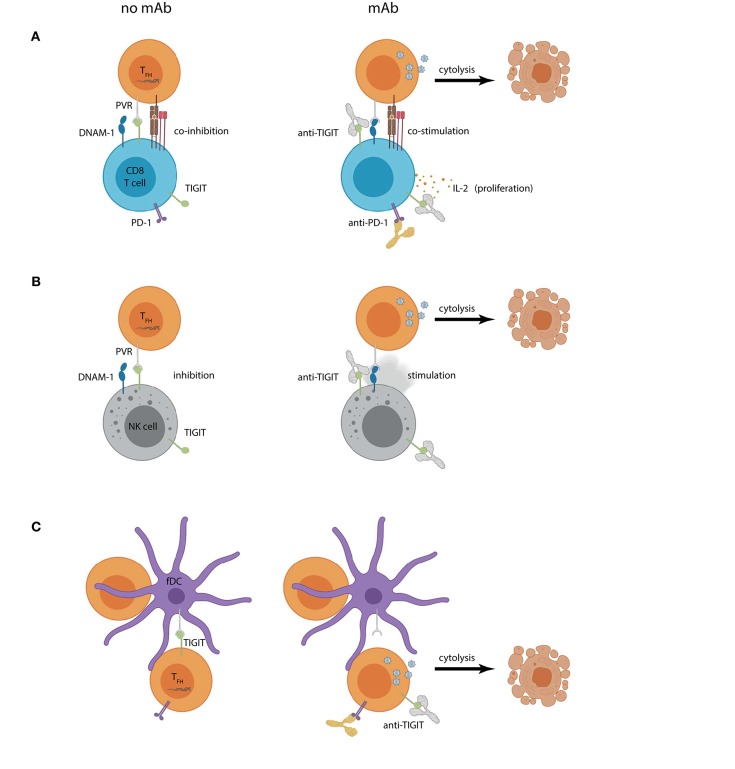
Hypothetical outcomes of using TIGIT blockade within a “shock and kill” approach for HIV-1 curative therapy. Increased PVR expression on lymph node CD4^+^ T_FH_ cells can contribute to **(A)** CD8^+^ T cell or **(B)** NK cell dysregulation by engaging TIGIT and (co)inhibiting effector functions (left panel). Combined CD4^+^ T cell reservoir activation and TIGIT mAb (right panel) could create a scenario where previously latent HIV-1 actively replicates, introducing targets for HIV-1-specific CD8^+^ T cell or NK cell recognition. Reservoir cytolysis is promoted in this scenario by preventing inhibitory TIGIT interactions and allowing DNAM-1 (co)stimulation. Combination TIGIT and PD-1 mAbs would also allow CD8^+^ T cell proliferation and IL-2 production. **(C)** Follicular DCs (fDCs) express PVR and interact closely with reservoir CD4^+^ T cells. TIGIT is also expressed on lymph node CD4^+^ T cells and may contribute to their suppression (left panel). Humanized anti-TIGIT mAb could aid in “shock”ing latent cells into productive infection by preventing CD4^+^ T_FH_ cell TIGIT interactions with PVR expressed on fDCs leading to virus-induced or effector cell mediated cytolysis.

Expression of TIGIT is broadly dysregulated on both CD8^+^ T cells and NK cells in HIV-1 infection. An increased fraction of CD8^+^ T cells expressing TIGIT arises despite early initiation of effective cART (Chew et al., 2016; Tauriainen et al., 2017). The high potential impact of targeting TIGIT as a therapeutic strategy to invigorate effector cell responses against HIV-1 is emphasized by TIGIT expression on more than half of CD8^+^ T cells and almost all HIV-1-specific CD8^+^ T cells in PLWH (Chew et al., 2016; Tauriainen et al., 2017). Cells expressing TIGIT proliferated less and mounted weaker antiviral cytokine responses compared with their TIGIT^neg^ CD8^+^ T cell counterparts, indicating a prominent role for TIGIT as a negative regulator of HIV-1-specific CD8^+^ T cell immunity (Chew et al., 2016). Additionally, TIGIT^pos^ CD8^+^ T cells from PLWH have increased PD-1 co-expression, which correlates with HIV-1 disease progression (Chew et al., 2016). ADDIN EN.CITE (Cella et al., 2010; Tauriainen et al., 2017; Yin et al., 2018) Interrupting TIGIT signaling using therapeutic mAb blockade rescues CD8^+^ T cell antiviral activity. If signaling through either TIGIT or PD-1 receptors is prevented by mAb, CD8^+^ T cell interferon (IFN)-γ responses and cytotoxicity increase (Johnston et al., 2014; Chew et al., 2016). However, IL-2 production and T cell proliferation is reestablished only when blockade of both receptors is imposed (Figure 2A) (Johnston et al., 2014; Chew et al., 2016). In parallel with increased TIGIT on CD8^+^ T cells, its costimulatory counterpart, DNAM-1, is often downregulated, further contributing to T cell exhaustion (Cella et al., 2010; Tauriainen et al., 2017). This “one-two punch” increases inhibitory intercellular TIGIT/PVR interactions and *cis* TIGIT/DNAM-1 heterodimers further restrict the potential for productive costimulation mediated by DNAM-1/PVR interactions (Figure 1).

Similar to the relationship seen with CD8^+^ T cells, higher levels of TIGIT on NK cells correlate with HIV-1 disease progression (Yin et al., 2018). Although TIGIT blockade can rescue NK cell function against cancer, further evidence illustrating the potential benefits of targeting the TIGIT axis in the context of HIV-1 infection is needed (Zhang et al., 2018). While TIGIT expression is increased on NK cells from treatment naïve PLWH, cART may return TIGIT expression to similar levels as that of healthy controls (Yin et al., 2018; Vendrame et al., 2020). In untreated PLWH, NK cells expressing higher amounts of TIGIT were less likely to degranulate and produce IFN-γ in response to cytokine stimuli than those that did not express TIGIT. In this case, baseline NK cell function was rescued by mAb against TIGIT (Yin et al., 2018). In another study in which NK cells were activated for 3 days with IL-2, blockade of TIGIT provided no benefits to NK cells responding against *in vitro* HIV-1 infected autologous primary CD4^+^ T cells (Vendrame et al., 2020). In the setting of active HIV-1 infection, TIGIT expression is increased on subsets of NK cells coexpressing DNAM-1 (Yin et al., 2018; Vendrame et al., 2020). Combining viral reactivation strategies with effector cell reinvigoration by preventing TIGIT interactions with either its ligand or DNAM-1 should promote cytolysis of infected cells (Figure 2B). More evidence is needed to delineate the cytotoxic potential of these cells. Expression of TIGIT on CD8^+^ T cells and NK cells suggests that TIGIT-specific mAb therapy could synergistically unleash both types of antiviral effector cells to more robustly target active HIV-1 infection.

### A Ligand for TIGIT Is Enriched on HIV-1 Reservoir Cells

Although expression levels of many inhibitory checkpoint molecules increase on multiple types of effector cells during HIV-1 infection, inhibition relies on the interactions between these receptors, and their cognate ligands. The predominant ligand for TIGIT and DNAM-1 is PVR, which is expressed on monocytes, dendritic cells, T cells and other cell types including tumor cells and HIV-1-infected cells (Mendelsohn et al., 1989; Pende et al., 2006; Chauvin et al., 2015; Chew et al., 2016). Originally identified in 1989 as a receptor for poliovirus, PVR belongs to a larger family of molecules that facilitate cell adhesion and migration, while over-expression of PVR in transformed cells promotes proliferation (Mendelsohn et al., 1989; Takai et al., 2008). Stimulated T cells have increased total PVR protein and cell surface expression levels, with preferential PVR expression on proliferating T cells in the S or G_2_/M cell cycle phase (Ardolino et al., 2011). Increased cellular PVR expression occurs after the DNA damage response (DDR) pathway is induced (Ardolino et al., 2011). Although activated primary CD4^+^ T cells express PVR, whether or not HIV-1 influences PVR expression on circulating primary CD4^+^ T cells remains controversial (Davis et al., 2017).

During infection, expression of HIV-1-encoded Vpr helps promote cell cycle arrest in G_2_ via the DDR pathway (Andersen et al., 2008). Through this same Vpr-dependent mechanism, PVR was reported to be upregulated on the surface of HIV-1-infected Jurkat T cells, yet expression of Nef and/or Vpu reduced surface-expressed PVR on both Jurkat and primary CD4^+^ T cells (Matusali et al., 2012; Vassena et al., 2013; Bolduan et al., 2014). Another study reported no role for HIV-1-specific modulation of PVR expression on primary CD4^+^ T cells (Davis et al., 2017). These studies used various *in vitro* systems with CD4^+^ T cell lines or *ex vivo* CD4^+^ T cells from healthy controls infected with different laboratory passaged HIV-1 strains. In all cases, PVR expression was assessed on all infected T cells, yet *in vitro*-infected CD4^+^ T cells can be subsequently distinctly grouped into either CD4^pos^ or CD4^neg^ cells (Tremblay-McLean et al., 2017). In so doing, Tremblay-McLean *et al*. found that surface PVR expression is reduced on infected CD4^neg^ T cells compared with infected CD4^pos^ T cells (Tremblay-McLean et al., 2017). This could indicate that if HIV-1 does regulate PVR expression *in vivo*, productively infected or reservoir T_FH_ cells that maintain their expression of CD4 may have a different PVR expression profile than their CD4^neg^ T cell counterparts.

Investigation of *ex vivo* PVR expression on CD4^+^ T cells from PLWH has been limited. Very low levels of PVR expression on circulating CD4^+^ T cells combined with the relative inaccessibility of lymph node sections from PLWH make informed assessment of PVR expression problematic (Yin et al., 2018; Vendrame et al., 2020). Upregulation of PVR can occur on CD4^+^ T cells in HIV-1 infection, especially on lymph node T_FH_ CD4^+^ T cells, which are the major site of HIV-1 reservoir concentration (Perreau et al., 2013; Banga et al., 2016; Tauriainen et al., 2017). Further, within the lymph nodes from PLWH, PVR is expressed on both germinal center CD3^+^ cells and interdigitating follicular DCs (Cella et al., 2010). This compact compartment comprised of cells expressing PVR in proximity to CD4^+^ T cells enriched in HIV-1 provirus could exploit higher localized TIGIT expression on CD8^+^ T cells and NK cells to limit effector cell functions as they transit through lymph nodes. As NK cell and CD8^+^ T cell expression of TIGIT increases with acute HIV-1 infection, introducing mAb therapy to overcome the higher affinity TIGIT/PVR inhibitory interaction in favor of DNAM-1/PVR-mediated activation is a rational strategy to address lingering HIV-1 infection (Yin et al., 2018). In this event, PVR expressed on reservoir CD4^+^ T cells would render them more susceptible targets for DNAM-1-expressing CD8^+^ T cells and NK cells (Figures 2A,B).

In 2019, killer cell immunoglobulin-like receptor (KIR)2DL5, an inhibitory receptor expressed on NK cells and CD8^+^ T cells, was identified as a binding partner for PVR, adding another facet to this already complex regulatory pathway (Estefania et al., 2007; Husain et al., 2019). The genes encoding KIR2DL5 (*KIR2DL5A* and *KIR2DL5*) are highly polymorphic (Vilches et al., 2000a,b). Less than 10% of CD56^dim^ NK cells and a very small fraction of the CD8^+^ T cells of carriers express the most common allele, 2DL5A^*^001, which is detectable by mAb UP-R1 (Estefania et al., 2007; Cisneros et al., 2012). An accurate measure of KIR2DL5 prevalence in the wider population is currently unavailable as it is unknown whether this is the only allele expressed or whether polymorphisms arising in other alleles alter epitopes recognized by UP-R1 (Cisneros et al., 2012, 2016). While multiple factors suggest that inhibiting TIGIT/PVR interactions is a suitable strategy to invigorate effector cell responses against HIV-1, PLWH expressing KIR2DL5 may be less likely to benefit from this approach. Studies are needed to determine the antiviral effector potential of NK cells expressing KIR2DL5 and whether they co-express other nectin or nectin-like receptors.

### TIGIT Is Expressed on HIV-1 Reservoir Cells

A significant hurdle to achieving HIV-1 cure is the lack of HIV-1 antigen expression on reservoir CD4^+^ T cells, which leaves no appropriate means to target them immunologically. Without specific cell surface markers or HIV-1 antigen expression/peptide presentation to identify HIV-1-infected cells, no level of competent effector cell function can eradicate HIV. Selectively targeting latently infected cells that comprise the HIV-1 reservoir is a subsidiary approach to HIV-1 cure. Together with CD8^+^ T cells and NK cells, CD4^+^ T cells, including T_FH_ cells residing deep within lymph node tissues, express TIGIT (Yu et al., 2009; Wu et al., 2016). The CD4^+^ T cell fraction expressing TIGIT is enriched for integrated HIV-1 DNA and the frequency of TIGIT^pos^ cells that also co-express PD-1 and LAG-3 correlates with the size of the HIV-1 reservoir (Fromentin et al., 2016). Expression of TIGIT on CD4^+^ T cells, alone or in combination with other immune checkpoint receptors identifies a subset of CD4^+^ T cells more likely to harbor latent HIV-1.

Although TIGIT expression can help identify HIV-1 reservoirs, these cells need to be activated or shocked into productive infection to express HIV-1 antigens or associated stress proteins enabling recognition by antiviral effector cells. Maintenance of stable HIV-1 reservoirs involves persistent inhibition through interactions between checkpoint inhibitors, such as PD-1 or TIGIT, and their ligands (Wykes and Lewin, 2018). Consistent with the latency reversal noted with anti-PD-1 mAb, introducing anti-TIGIT mAb to unleash negative regulation can help shock TIGIT-expressing CD4^+^ T cells into activation and shift latent HIV-1 into active production (Figure 2C) (Chew et al., 2016; Fromentin et al., 2016, 2019; Evans et al., 2018; Guihot et al., 2018; Wykes and Lewin, 2018). Targeting TIGIT as part of a cure strategy for HIV-1 could concurrently help force HIV-1 out of hiding, while rescuing CD8^+^ T cell and NK cell antiviral functions to bridge effector cell functions with recognition of HIV-1-infected cells–a multipronged “shock and kill” approach.

## Conclusion

We have discussed findings that suggest TIGIT inhibition of CD8^+^ T cell and NK cell surveillance against HIV-1-infected CD4^+^ T cells and monocytes, compounded by dysregulation of PVR and DNAM-1 expression, constitutes an exploitable immune checkpoint in HIV-1 reservoir elimination strategies engaging antiviral effector cells. The reasons that TIGIT could be an especially attractive target are several fold. Most importantly, TIGIT is expressed on most NK cells and almost all HIV-specific CD8^+^ T cells in PLWH (Wang et al., 2015; Tauriainen et al., 2017; Yin et al., 2018). While this may favor targeting TIGIT over other inhibitory receptors such as PD-1 or CTLA-4, there is a case for using combinations of checkpoint inhibitors. For example, blocking TIGIT increased degranulation, IFN-γ production and proliferation of antiviral effectors, however, blockade of both TIGIT and PD-1 rescued IL-2 production, an important correlate of immune stability in PLWH (Johnston et al., 2014; Chew et al., 2016). In some settings, TIGIT blockade increases NK cell natural degranulation and antiviral cytokine release and likewise enhances cytokine release by CD8^+^ and CD4^+^ T cells and degranulation of HIV-specific CD8^+^ T cells (Chew et al., 2016; Fromentin et al., 2016; Tauriainen et al., 2017; Yin et al., 2018). Secondly, there is evidence that HIV-1 infection of CD4^+^ T cells induces upregulation of PVR expression and that the latent HIV reservoir is to some extent concentrated in CD4^+^ T cells expressing PVR and/or TIGIT (Cella et al., 2010; Tauriainen et al., 2017; Yin et al., 2018). Thus, at least a fraction of the CD4^+^ T cells activated for nascent HIV-1 replication is pre-armed to inhibit antiviral effector cell function through PVR engagement of TIGIT. Previous studies indicating that endogenous HIV-specific CD8^+^ T cell responses of PLWH are insufficient to address nascent HIV-1 reactivation underscore the necessity to enhance antiviral effector functions in concert with HIV-1 reactivation (Shan et al., 2012).

The breadth of its effects on T cells and NK cells as well as specificity for cells in which the HIV-1 reservoir is concentrated combine to highlight the potential of TIGIT blockade in immunotherapeutic HIV-1 cure strategies. Several humanized anti-TIGIT mAb (AB154 and Etigilimab) have already entered clinical trials in cancer therapy, alone and in combination with anti-PD-1. These early stage studies indicate a favorable safety profile with effective TIGIT blockade. Experience in the cancer setting should help inform strategies for TIGIT blockade in PLWH, including whether better outcomes can be achieved when used in combination with other checkpoint inhibitors. For cure strategies that involve widespread reactivation of HIV replication and purging of the exposed infected cells, it will be critical to determine which effector cells or functions can most rapidly be brought to bear against nascent HIV-1 replication.

## Author Contributions

KH and MG jointly wrote and edited the manuscript.

## Conflict of Interest

The authors declare that the research was conducted in the absence of any commercial or financial relationships that could be construed as a potential conflict of interest.
